# Cobot selection using hybrid AHP-TOPSIS based multi-criteria decision making technique for fuel filter assembly process

**DOI:** 10.1016/j.heliyon.2024.e26374

**Published:** 2024-02-15

**Authors:** Sivalingam C, Senthil Kumar Subramaniam

**Affiliations:** School of Mechanical Engineering, Vellore Institute of Technology, Vellore, 632014, India

**Keywords:** Collaborative robot, Assembly task, MCDM, AHP, TOPSIS

## Abstract

The choice of a suitable collaborative robot (cobot) for a real-time industrial process is one of the obstacles to effective robot implementation in terms of energy and cost. The cobot selection process for an application have become more complex due to increasing sophisticated features and capabilities in cobots offered by the manufacturers. The paper presents a hybrid Multi-Criteria Decision-Making (MCDM) technique based on Analytical Hierarchy Process (AHP) and Technique for Order of Preference by Similarity to Ideal Solution (TOPSIS) approaches for selecting cobots for fuel filter assembly operation. The product design methodology, manufacturing method, and associated cost are directly influencing the decision on cobot selection. The most appropriate robot to accomplish the desired task at the lowest possible cost and capability can be selected by AHP with prospective criterion weight for subsequent processing. The TOPSIS approach orders alternatives based on the prominence of criteria. A diesel fuel filter assembly process case was considered for validating the proposed technique of cobot selection process. An expert knowledge base was generated for 12 advocated cobots and the best cobot was selected using the proposed hybrid MCDM technique.

## 1 Introduction

Collaborative robots are multipurpose, reprogrammable devices with specific anthropometric characteristics. The decision-making ability, capacity to respond to different sensory inputs, and ability to communicate with other machines, make the cobot an essential tool for a variety of industrial applications, including material handling, assembly, finishing, machine loading, spray painting, and welding. When choosing industrial cobot for a specific application, the most important factors to be taken into account are control resolution, accuracy, repeatability, load-carrying capacity, degrees of freedom, man-machine interfacing ability, programming flexibility, maximum tip speed, memory capacity, and supplier's service quality. These influencing factors can be divided into objective and subjective factors or advantageous and disadvantageous factors. The cost of a cobot and its ability to carry a load are objective characteristics that can be quantified statistically. On the other hand, subjective attributes are qualitative, such as level of service by vendor, robot's programming flexibility, etc.

The load-carrying capacity, programming flexibility, and non-benefit features, such as cost and repetition, must be considered when choosing industrial cobot for a specific application. The trade-off between the robot features and Cabot performance metrics is vital to select cobot with multi-criteria decision-making (MCDM) techniques. A standard methodology for determining cobots for any application needs to be improved due to complexity in robot selection with varying characteristics [1]. The industrial and service sectors use cobot for tasks in their specific work environments, which impacted by lack of a consistent process for cobot selection. Services providers developed an interest in an automated solution for the deployment of cobots for a particular task in the workplace. Researches are carried out to deliberate an industry-wide standard technique for choosing the best cobot for any application [2].

The present work addresses the need for a standard selection method for cobot and to compile a comprehensive list of cobot attributes and criteria and to apply an algorithm to the problem of cobot selection. The Analytical Hierarchy Process (AHP) and Technique for Order of Preference by Similarity to Ideal Solution (TOPSIS) were used as the hybrid MCDM algorithm to address the cobot selection problem, which was not experienced for cobot selection for an assembly process.

## 2 Multi-criteria decision-making

Industrial robots have varied capabilities and requirements for an application; due to the wide variety of robots on the market, it's difficult to choose suitable robot for a specific industrial requirement. Multi-Criteria Decision Analysis (MCDA) techniques are frequently employed in all the fields of research. A lot of research had been carried out on the advancement of MCDA techniques and less attention has been given to decision problems [3]. The MCDA techniques allowed the decision-makers' preferences for relative weighting of robot selection attributes. A modified Multi Attribute Decision Making (MADM) method was attempted to check the variation in ranking performance with the change in normalization technique [4].

The relative performance and rankings of robots performing industrial pick & place operations by MCDM methods concluded that finding the best MCDM approach need to consider the correct criteria and options for a specific industrial robot [5]. PROMETHEE II method was exercised as an efficient decision-making techniques for complete ranking order of all available alternatives and leads to error free decision making [6]. Weighted Sum Method (WSM), Weighted Product Method (WPM), Weighted Aggregated Sum Product Assessment (WASPAS), Multi-Objective Optimization on the basis of Ratio Analysis and Reference point Approach (MOORA), and Multiplicative Form of MOORA method (MULTIMOORA) are well-known and easy to use MCDM techniques [7]. Evaluated Based on Distance from Average Solution (EDAS) is an efficient MCDM method to the robot selection problem (RSP). When comparing the results of EDAS with other approaches used for grading industrial RSPs, Spearman's Rank Correlation Analysis demonstrated that EDAS is capable of accurately rating selected robots [8]. The industrial robot selection problem was addressed by employing the Fuzzy Best-Worst and PROMETHEE methods in MCDM for weighing criteria and ranking decision alternatives, respectively [9]. A combination of three MCDM approaches, Fuzzy versions of AHP, TOPSIS, and Simple Multi Attribute Rating Technique (SMART) was exercised for selecting an industrial robot for a universal, flexible assembly station based on technical and performance aspects. The analysis results and final categorization based on decision makers' preferences for robot parameters were created to decrease the method's impact on the final decision [10]. The best-least approach to determining criteria weights may be related to the assessment based on EDAS method, which was more relevant and requires less computation compared to other approaches [11].

Chodha et al. (2021) investigated a method to select the most suitable industrial robot for arc welding process with TOPSIS and Entropy MCDM techniques from eight robot options and five criteria. The objective significance weights of traits and criteria were assigned using entropy weights approach [12]. The combined fuzzy SWARA and fuzzy CoCoSo with Bonferroni function was developed to choose the best industrial robot for use in automotive industry. As a result of the investigation, the most crucial parameters, precision, reach and performance had been determined. To verify the findings, a detailed sensitivity analysis was conducted. The results were compared with similar decision making techniques. The outcomes of the analyses for sensitivity confirm the applicability and reliability of the proposed model [13]. An industrial robot selection problem utilising TOPSIS-ARAS and COPRAS-ARAS hybrid MCDM systems, which greatly improved the prior recommended rankings by reducing complexity, confusion, and vagueness [14]. Integrated Weight Assessment Ratio Analysis (SWARA) and Combined Compromise Solution (CoCoSo) techniques were employed to determine suitable spray painting robot for automotive [15].

The selection criteria for cobot with updated multi-criteria decision-making algorithm was demonstrated to choose an appropriate cobot for manufacturing or service industries [16]. The common MCDM methods used to solve the industrial cobot selection; AHP, TOPSIS, VIKOR, ELECTRE II, PROMETHEE, and DEA are listed in Table 1.

**Table 1:** MCDM approaches for industrial robots selection.

References	Criteria	Weight measurement	MCDM techniques	Application
[17]	Maximum tip speed, repeatability, memory capacity, manipulator reach, payload, velocity, cost, vendor's service level.	AHP	VIKOR	Pick-*n*-place operation
[18]	Programme adaptability, vendor's training, velocity, load capacity, and vertical reach, repeatability error.	–	FUZZY - TOPSIS	Assembly
[19]	Tool speed, Cost, coefficient of handling payload, repeatability, velocity, memory capacity, and arm reach.	AHP	WSM, WPM, TOPSIS, VIKOR, PAM	Machine support, Handling operation
[20]	Load capacity, reach, weight, repeatability, power consumption, customer service	AHP	AHP	Milling
[21]	Operation performance, maintenance, axis movement reach, vertical movement, Payload repeatability, weight, power rating, price flexibility, and safety.	Entropy	MABAC	Arc welding
[22]	Manipulator reach, velocity, pay load capacity, cost, repeatability, maximum tool speed, and memory capacity.	CRITIC	TOPSIS-ARAS, COPRAS-ARAS	Pick-and-place operation
[23]	Degree of freedom Load capacity, repeatability, and velocity ratio.	BWM	EDAS	Manufacturing
[24]	performance, flexibility, purchase cost, maintenance cost, operation time, operating cost, working accuracy, warranty period, arm use, and load capacity.	Fuzzy SWARA	Fuzzy CoCoSo	Manufacturing
[25]	Mechanical weight, repeatability, payload, maximum reach and average power.	ENTROPY	TOPSIS	Industrial application
[26]	Payload, mechanical weight, speed, repeatability, reachability, cost and power and consumption.	CoCoSo	SWARA	Manufacturing
[27]	Payloads, speed, reach, mechanical weight, repeatability, cost and power consumption.	CODAS, COPRAS,	MEREC	Industrial application
[28]	Economic cost, load capacity, repeatability error, top speed, kinematic structure and vertical reach, horizontal reach, memory capacity, weight and power consumption.	SAW	TOPSIS, VIKOR, ELECTRE III	Industrial automation
[29]	Type of drives, memory capacity, maximum tip speed, load capacity, manipulator reach, and repeatability.	COPRAS	fuzzy COPRAS, WASPAS	Manufacturing
[30]	Performances, flexibility, purchase cost, Maintenance cost, operating cost, working accuracy, warranty period, arm use, and load capacity.	Fuzzy SWARA, fuzzy CoCoSo.	Bonferroni function	Manufacturing
[31]	Performance, flexibility, purchase cost, maintenance cost, working accuracy, warranty period, load capacity, and energy consumption.	Entropy	MOORA	Automotive industry

From the literature on MCDM approaches for selection of industrial robot, it was observed that the cost and safety criteria were not addressed adequately. The proposed MCDM-based algorithm for the cobot selection process is a combined method to find out criteria weight using AHP and to evaluate the ranking of cobots using TOPSIS method for flexible assembly station, considering payload, repeatability, reachability, speed, weight, power, and cost. The proposed technique is a straight approach for cobot selection problem and combines AHP and TOPSIS as hybrid MCDM method for weighing criteria and ranking decision alternatives to overcome uncertainties in the decision-making process for cobot selection. The present work intends to develop a hybrid decision-making method to identify an appropriate collaborative robot for the filter assembly process by integrating multiple robot parameters.

## 3 Methodology

In this work, a combined Analytical Hierarchy Process (AHP) and Technique for Order of Preference by Similarity to Ideal Solution (TOPSIS) approach is suggested for selecting suitable industrial cobot for an industrial assembly process. A straightforward, dependable, and robust MCDM approach is exercised for selecting industrial cobot based on their parameters. Consistent findings, fewer pairwise calculations, and ease in choosing the best and worst criteria compared to other measures are benefits of using the AHP approach for weight calculations. To rank the robots, the TOPSIS technique was chosen since, compared to other MCDM methods as it needs the least processing.

### 3.1 Analytical hierarchy process

**Fig. 1:**
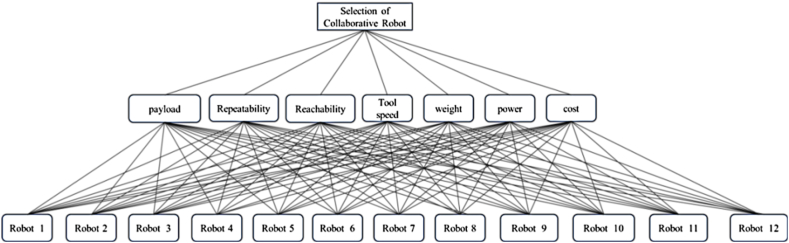
AHP structure for cobot selection process.

The analytical hierarchy procedure is used in MCDM technique to solve the problems in cobot selection and the proposed cobot selection method's hierarchical structure is shown in Fig. 1.

AHP undergoes through several stages, including ranking difficult decision-making issues and giving more weight to the criteria that support alternative priorities investigated [32]. The stages are.

1. Identification of the problem.
2. Construction of hierarchy of goals, factors influencing behaviour, and potential solutions for the problem.
3. Pairwise Comparison Matrix (PCM) generation utilising Table 1 and advice from experts. The level of each component on the numerical scale is determined through comparison. It takes [*n(n-1)/2*] comparisons, where ‘*n*' is the number of criteria and the diagonal elements are either equal to 1 or not equal to 1, and the remaining details are the reciprocals of the preceding comparisons which form the matrix represented by Eq. (1).

(1) $$A=\left[\begin{array}{cccc} 1 & a_{12} & \cdots & a_{1n}\\ {1/a}_{12} & 1 & \cdots & a_{2n}\\ \vdots & \vdots & 1 & \cdots \\ {1/a}_{1n} & {1/a}_{2n} & \cdots & 1 \end{array}\right]$$

### 3.2 Technique for order of preference by similarity to ideal solution

The Multi-Criteria Decision-Making (MCDM) methodology based on Technique for Order Preference by Similarity to Ideal Solution (TOPSIS) method is used to select an industrial robot for assembly operations. The Entropy weight method is used to obtain the weights of significance using objective preferences. The TOPSIS-Entropy technique's results of the ranking are provided in sequence. The steps for TOPSIS ranking computation are.

(i) To determine the output parameters, the initial decision matrix using the cobot experimental output data was created.
(ii) The normalised values of the first choice matrix were constructed with Eq. (4).

(4) $${\;z}_{ij}=\frac{X_{ij}}{\sqrt{\sum_{k=1}^{n}x_{kj}^{2}}}$$

Where z_ij_ is the normalised value of the experimental data.

(iii) The weight (w_i_) was computed and multiplied by the normalised evaluation matrix (zij) to create the weighted normalised decision matrix using the following Eq. (5).

(5) $${wn}_{ij}=w_{i}*\;z_{ij}$$

(iv) The Positive Ideal Solution (PIS), *A*_i_ + as the most desirable alternatives and Negative Ideal Solution (NIS), *A*_*i*_^*-*^ as the least desirable alternatives are computed by Eq. (6) and Eq. (7).

(6) $$A_{i}^{+}=\left\{A_{1}^{+},\ldots \ldots .\;A_{i}^{+}\right\}=\{\max_{j}A_{\text{ij}}|i\in I^{\prime }\},\{\min_{j}A_{\text{ij}}|i\in I^{\prime \prime }\}\text{,}$$

(7) $$A_{i}^{-}=\left\{A_{1}^{-},\ldots \ldots .\;A_{i}^{-}\right\}=\{\min_{j}A_{\text{ij}}|i\in I^{\prime }\},\{\max_{j}A_{\text{ij}}|i\in I^{\prime \prime }\}\text{,}$$

(v) The distance parameters in Euclidean space *D*_*i*_^*+*^ and *D*_*i*_^*-*^ are computed by Eq. (8) and Eq. (9).

(8) $$D_{i}^{+}=\sqrt{\sum_{j=1\;}^{n}{\left(A_{ij}-{A_{j}}^{+}\right)}^{2}}$$

(9) $$D_{i}^{-}=\sqrt{\sum_{j=1\;}^{n}{\left(A_{ij}-{A_{j}}^{-}\right)}^{2}}$$

(vi) The distance coefficient *CC*_i_ to the ideal solutions is computed using Eq. (10).

(10) $${CC}_{i}=\frac{D_{i}^{-}}{D_{i}^{-}+D_{i}^{+}}$$

### 3.3 Hybrid AHP-TOPSIS technique

**Fig. 2:**
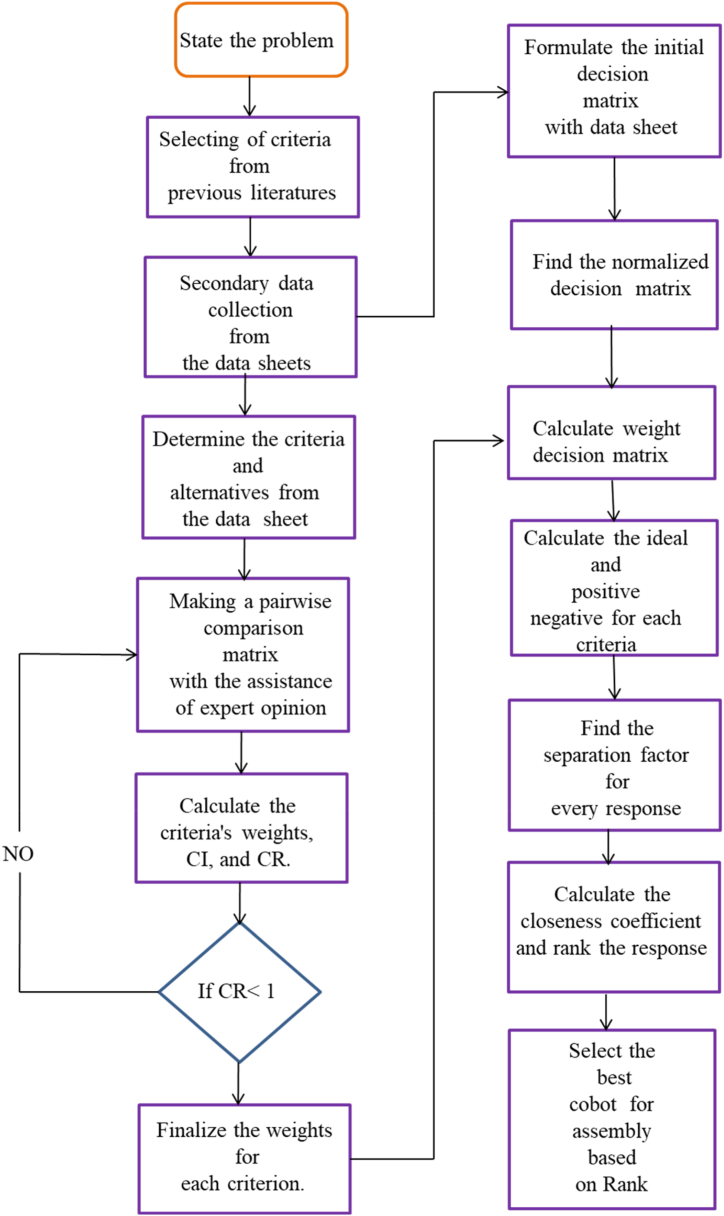
Hybrid AHP-TOPSIS decision making methodology.

The AHP-TOPSIS hybrid techniques with weighing and decision-making methods produce the ranking of cobots. During the weighting process, the AHP approach is used to reduce the subjectivity of the decision-makers. The concepts' clarity, simplicity, computational effectiveness, and speed attribute to the TOPSIS approach ideal for selection [12]. The AHP-TOPSIS analysis sequential procedure is presented in Fig. 2. The initial stage consists of integrated system analysis. The information required for hybrid analysis has been acquired. An expert knowledge is used to evaluate each of these configurations, since it directly affects the results. The expert opinion was used to construct pairwise comparison matrix. The hierarchy presented in the AHP analysis determines the criteria weights of the performance and emission requirements, which stands for the robot cost, repeatability, reachability, tool speed, weight, power consumption, and payload. The weights of TOPSIS's criteria were determined by the outcomes of the AHP stage. In order to achieve the hybridization of AHP and TOPSIS, the weights were included in the analysis.

## 4 Selection of cobot for fuel filter assembly - case study

**Fig. 3:**
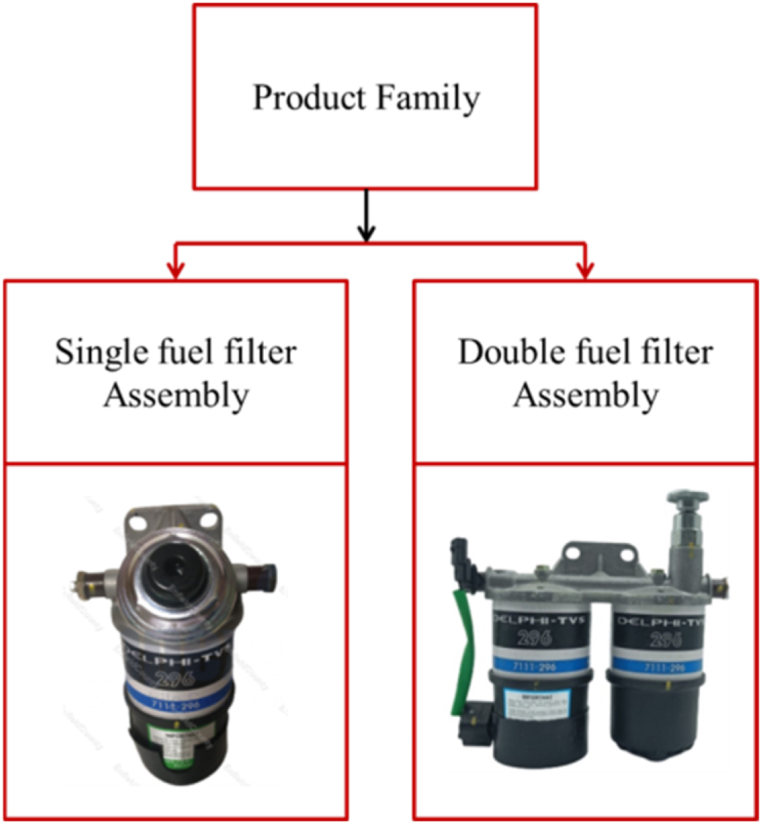
Part family of fuel filter.

Mostly, the manufacturing process is either partially or totally automated, but most of the assembly processes are still in manual mode. Due to the complexity of mechanical systems, such as fuel filter assembly, manual assembly is still widespread in the automobile industry. Often, the assembly of such elements is too complicated to be fully automated. The fuel filter component has complex assembly and comparatively high number of different components. It comprises a filter head, plug, inner tube, bowl, bleeder screws, gasket, etc. An organised strategy for creating human-robot work teams based on the complementary skill sets can assess the complexity of an assembly operation and then assigning duties to humans and robots. The method integrates the safety in human-robot assembly as well as the dynamics of the Human-Robot Collaboration (HRC) environment, such as part presentation and feeding, in addition to the geometrical and physical features of assembly components, which are listed in Table 4. Fig. 3 illustrates the part family of fuel filter used in the present study.

**Table 2:** Scale of relative alternatives (Saaty scale).4.Computation of the normalization and criteria weights of the pairwise comparison matrix.5.Determination *λ*_max_ is the principal Eigen value, a global summation of the product of the sum of each vector column for both decision matrix and pairwise comparison matrices and computed with the pair wise values for each row.6.Consistency Index (CI) is computed by Eq. (2).(2)$CI=\frac{\lambda \;\max-\;n\;}{n-1\;}$7.Consistency Ratio (CR) is computed by Eq. (3).(3)$CR=\frac{CI}{RI\;}$8.Random consistency Index (RI) is chosen according to the number of criteria as listed in Table 3.

Performance intensity value	Description
1	Identical significance
3	Greater significance
5	Much more crucial
7	Significantly more crucial
9	Unquestionably more crucial
2, 4, 6, 8	Values in the middle

**Table 3:** Random index (RI).9.If the calculated CR is less than 0.10, then the expert evaluations are assumed to be consistent and the weight findings are accepted. The procedure is repeated until these values fall below the permissible range.

No. of Criteria	1	2	3	4	5	6	7	8	9	10
Random Consistency Index	0	0	0.58	0.9	1.12	1.24	1.32	1.41	1.45	1.49

**Table 4:** HRC automation for fuel filter assembly.

S. No.	Components	Task allocation
1	Glue Application- Center Tube	Human Operator
2	Coil Winding	Human Operator
3	Inspection	Human Operator
4	Holes Gap	Human Operator
5	Sizing	Human Operator
6	Seaming	Human Operator
7	Drying	Human Operator
8	Printing	Human Operator
9	Filter Assembly	Cobot
10	Marking	Human Operator
11	Vacuum Support & Plug Assembly	Human Operator
12	Leak Test	Human Operator
13	Visual Test	Human Operator
14	Packaging	Human Operator

## 5 Data aggregation

The websites and manuals of the relevant cobot manufacturers were used as secondary data sources to acquire details on the technical characteristics of the collaborative robots under consideration [28]. The data are often compiled and summarised to increase the effectiveness of research. There are 12 suitable robot models from various manufacturers were considered for the intended application and the AHP-TOPSIS hybrid MCDM method is exercised in solving cobot selection challenge. The cobot characteristics were selected as comparison criteria, which are listed in Table 5. The listed cobots possess six degrees of freedom. The cobot operational performance was assessed based on the evaluation criteria, i.e. Payload (C1), Repeatability (C2), Reachability (C3), Tip speed (C4), Mechanical weight (C5), Power consumption (C6), and Robot cost (C7). The positive traits C1, C3, and C4 are expected to have greater values, while the negative attributes C2, C5, C6, and C7 have lower values.

**Table 5:** Criteria for cobot selection.

S. No.	Criteria	Definition
1	Payload in Kg (C1)	The maximum weight the cobot can lift.
2	Repeatability in mm (C2)	An evaluation of the robot's reliability in achieving a given goal.
3	Reachability in mm (C3)	It is the maximum distance between the end effector-holding component's maximal extension and the canter of the robotic structure.
4	Tool speed in m/s (C4)	Speed mostly depends on the cycle time required to execute the work, considering the robot's proper acceleration and deceleration as it moves from point A to point B.
5	Weight in Kg (C5)	Defined as the robotic structure's overall weight.
6	Power in W (C6)	The entire amount of power needed by the robotic structure.
7	Cost in INR (C7)	Cost relates to the overall cost spent on buying a collaborative robot for a particular assembly operation.

Commonly, the robot repeatability range between 0.02 mm and 0.05 mm is deemed to be optimal. If the application process has repetitive tasks, such as packaging, palletizing, etc. then it needs to be automated. Such cases, the collaborative robot does not need to be extremely accurate, but repeatability is preferred. The distance between cobot centre and its furthest arm extension is termed as reach and it serves to identify the work envelope of a cobot. Power consumption is the total amount of energy required to operate cobot, whereas cost criterion is the overall cost of robot system for a specific assembly process. The less power consumption of cobot system results in overall cost savings. To implement the cobot selection strategy, medium-sized serial industrial cobots were identified that can carry out assembly tasks with specific components. The criteria information on twelve collaborative robot alternatives are presented in Table 6.

**Table 6:** Cobot alternatives for diesel fuel filter assembly.

S. No.	Collaborative Robot	Criteria						
		Payload (Kg)	Repeatability (mm)	Reachability (mm)	Speed (m/s)	Weight (kg)	Power (W)	Approximate Cost (INR)
1	UR3	3	0.03	850	1	18.4	200	2148133
2	JAKA Zu 3	3	0.02	626	1.5	12.0	150	1239308
3	AUBO Robotics i3	3	0.03	625	1.9	15.5	150	1652410
4	Elite Robots CS63	3	0.02	624	2	16.5	150	1652410
5	Dobot CR3	3	0.02	624	2	16.5	150	1651584
6	EVC 3	3	0.02	620	1	15.0	120	1404549
7	Elibot EC63	3	0.02	620	1	15.0	120	1083981
8	Elephant Robotics Panda 3	3	0.05	550	1	17.0	260	1569707
9	Frank Emicka Panda	3	0.10	855	1	17.8	80	1074067
10	Hanwha HCR-3	3	0.05	630	1	13.0	240	1817651
11	Precision Automation PF3400	3	0.02	590	2	18.0	100	1652410
12	Elfin 6-axis collaborative robot	3	0.02	590	2	18.0	100	1652410

### 5.1 Criteria weight

The Analytic Hierarchy Process (AHP) method estimates subjective weights of the factors under consideration. The criteria were sorted according to the predicted relative significance based on group of consensus on achieving the primary needs of the assembly operation by decision-makers/experts. After consultation with experts, the relative importance of consideration criteria were assigned through conversation and brainstorming. The collaborative robot's acquisition cost is given the most weight among these factors, following the payload capability. The cobot mechanical weight is assumed as least importance. As a result, the ranking of the evaluation parameters is set to cobot cost, payload, power consumption, repeatability, reachability, tool speed, and mechanical weight. The Pairwise Comparison Matrix (PCM) as shown in Table 7 is executed using the Saaty technique [33] using scale of relative alternatives presented in Table 2. The outcome of the AHP computations of the criteria weights are shown in Table 8.

**Table 7:** Pairwise comparison matrix.

Robot Parameters	Robot Cost	Repeatability	Reachability	Tool Speed	Weight	Power Consumption	Payload
Robot Cost	1	1/2	3	9	3	1/2	4
Repeatability	2	1	3	1/2	2	1/2	2
Reachability	1/3	1/3	1	1/2	4	1/2	2
Speed	1/9	2	2	1	2	9	2
Weight	1/3	1/2	1/4	1/2	1	1/2	1
Power Consumption	2	2	2	1/9	2	1	2
Payload	1/4	1/2	1/2	1/2	1	1/2	1

**Table 8:** AHP results.

Criteria	Criteria Weight	AHP Parameters	Consistency ratio
Robot Cost	0.193691277	*λ*_max_ = 7.517657621	0.1
Repeatability	0.168731748	CI = 0.08627	
Reachability	0.146183274	RI = 1.32	
Speed	0.164338082		
Weight	0.097435230		
Power Consumption	0.156558880		
Payload	0.073061510		

The consistency index and consistency ratio of the chosen criteria from the pairwise comparison study were calculated using Eq. (2) and Eq. (3), and the results are presented in Table 8. The relative criteria weights were determined by applying Eq. (5). It can be seen that robot cost has the highest weight in the specified priority order of criteria weight. The cobot payload and weight are the least important criteria based on criteria weight computed by AHP.

### 5.2 Ranking by technique for order of preference by similarity to ideal solution

**Fig. 4:**
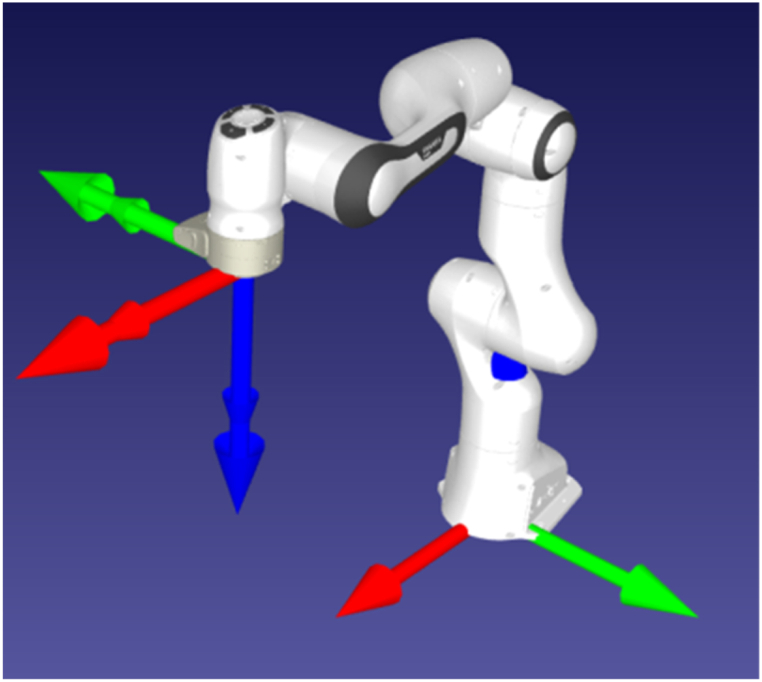
Frank Emicka Panda cobot.

The data from Table 6 fed as the initial decision matrix conferring to TOPSIS. Based on the assessment criterion employed in the collaborative robot selection problem, all elements were linearly normalised in order to transform them into a range of 0–1. Eq. (6) and Eq. (7) were used evaluate the A_i_^+^ and A_i_^−^, Eq. (8) and Eq. (9) were used to compute total Euclidean distances (D_i_^+^, D_i_^−^). The distance coefficients to the ideal solutions (CCi) were computed with Eq. (10). The derived final rankings of all cobot alternatives are listed in Table 9. The final appraisal ratings were subsequently determined by considering estimates of expert's scores. The Frank Emicka Panda robot as shown in Fig. 4 was determined to be the most suitable cobot for the fuel filter assembly tasks by the proposed hybrid MCDM technique. The cobot specification are listed in Table 10.

**Table 9:** Ranking of cobot alternatives using AHP-TOPSIS.

Alternatives	Most Desirable Alternatives A_i_^+^	Least Desirable Alternatives A_i_^-^	Distance Coefficient CC_i_	Rank
AUBO Robotics i3	0.089702246	0.102614981	0.533571446	5
Dobot CR3	0.097175251	0.117107006	0.546508178	2
Elephant Robotics Panda 3	0.092517936	0.100754951	0.521309289	9
Elfin 6-axis collaborative robot	0.100477583	0.116116598	0.536102113	3
Elibot EC63	0.085419426	0.091718396	0.517779856	11
Elite Robots CS63	0.101132006	0.116291976	0.534862690	4
EVC 3	0.103463213	0.113945430	0.524107175	8
Frank Emicka Panda	0.012214060	0.088876878	0.879177498	1
Hanwha HCR-3	0.083638095	0.090287280	0.519115051	10
JAKA Zu 3	0.099979666	0.112180896	0.528754709	6
Precision Automation PF3400	0.130012072	0.143420503	0.524518715	7
UR5	0.113285192	0.120433302	0.515292137	12

**Table 10:** Specifications of Frank Emicka Panda cobot.

Cobot Model	Repeatability (mm)	Reachability (mm)	Speed (m/s)	Weight (kg)	Power (W)	Payload (kg)
Frank Emicka Panda	0.1	855	1	17.8	80	3

## 6 Conclusion

Collaborative robots (cobots) are engaged in industrial automation due to their capability in carrying out repetitive, hazardous, and difficult tasks in a safe manner. The decision becomes considerably more difficult to choose appropriate cobot model within their capabilities to complete activities in an economic way. In the present work a hybrid MCDM technique was executed in selection of best cobot alternative for an industrial assembly operation. The AHP approach was applied to determine the prospective criteria weights and TOPSIS method ranked the alternatives based on the weight of the criteria. The presented MCDM technique has been validated by selecting a collaborative robot for a fuel filter assembly process from twelve alternative cobot choices with seven criteria. Considering the technical and operational parameters, the Frank Emicka Panda cobot was determined to be prominent for the fuel filter assembly workstation using the proposed hybrid multi-criteria decision making methodology. Implementing the best cobot could reduce overall time of fuel filter assembly process and leads to an economic operation. The proposed hybrid MCDM method is straightforward, methodical, easily accessible, and simple to incorporate into cobot decision-making process. Compared to the other methods, the proposed hybrid MCDM technique is complete, reliable, and offers a true ranking order. Future research in the task allocation problem with implementation of appropriate collaborative robot selection can enhance the assembly process optimization.

## CRediT authorship contribution statement

**Sivalingam C:** Conceptualization, Formal analysis, Methodology, Writing – original draft. **Senthil Kumar Subramaniam:** Conceptualization, Methodology, Resources, Supervision, Writing – review & editing.

## Declaration of competing interest

The authors declare that they have no known competing financial interests or personal relationships that could have appeared to influence the work reported in this paper.
